# Structural and Tribological Characterization of Carbon and Glass Fabrics Reinforced Epoxy for Bushing Applications Safety

**DOI:** 10.3390/polym15092064

**Published:** 2023-04-26

**Authors:** Majed H. Moosa, Mohamed Abu-Okail, Ahmed Abu-Oqail, Samah A. Al-Shelkamy, W. M. Shewakh, M. Abdel Ghafaar

**Affiliations:** 1 Department of Industrial Engineering, Faculty of Engineering, Jazan University, Jazan 82817, Saudi Arabia; mmoosa@jazanu.edu.sa (M.H.M.); waleedshewakh@hotmail.com (W.M.S.); 2 Manufacturing Engineering and Production Technology Department, Modern Academy for Engineering and Technology, Cairo P.O. Box 11571, Egypt; dr.metwally123@gmail.com; 3 Mechanical Production Department, Faculty of Technology and Education, Beni-Suef University, Beni-Suef P.O. Box 62521, Egypt; ahmed_abuoqail@yahoo.com; 4 Physics Department, Faculty of Science, New Valley University, El-Kharga P.O. Box 72511, Egypt

**Keywords:** carbon-and glass-fiber-reinforced polymers, microstructural characteristics, wear resistance, bushing applications

## Abstract

This article investigates the effect of geometrical alternatives for fiber directions on the structural and tribological properties of glass and carbon fibers when molded with epoxy as polymeric composite fabrics for the safety and quality of bushing applications. To confirm the best composite fabric direction, scanning electron microscope and tribological analyses were carried out for the glass and carbon fabrics at horizontal and vertical geometrical alternative orientations. The tribological test was applied using a pin-on-disk tribometer at constant bark velocity of 0.520 m/s against different loads, beginning with 5, 10, 15, and 20 N for the investigated composite samples. The structural measurements demonstrated that the carbon fiber had a high ability to merge with the resin epoxy when compared with the glass fiber. The tribological analysis elucidated that the lower wear volume loss and friction coefficient were obtained when molding the resin epoxy horizontally to the fiber-stacking direction compared with the other vertical direction. Accordingly, the study deduced that the carbon fiber composite material achieves superior wear resistance when molded by resin epoxy horizontally to the direction of tribological wear, which is suitable for several advanced bushing applications.

## 1. Introduction

Polymers are multifunctional structural substances; they are composed of several microstructural units assembled by the same type of linkage [1,2,3,4,5] and demonstrate better functionality and safety when compared with machining by other materials separately [6,7,8]. Polymers are in great demand in several industrial technological engineering and economical applications [9,10].

Polymer matrix composites are extensively used in modern industrial applications, such as the electronic, aerospace, and automotive industries [11]. They have multiple benefits: their light weight, good strength, and a relatively inexpensive cost, as well as an extraordinary thermal stability, damping resistance, and corrosion resistance [12].

Some of the most famous types of polymer matrix composites are fiber-reinforced polymers [13,14,15]. They have good mechanical properties, including their elastic modulus and toughness [16]. Fiber-reinforced polymers are used in several sectors, including the aviation, electrical power, marine [17,18,19,20,21,22], railway [8,23,24], and textile industries [25].

Lightweight fiber materials are classified into glass, carbon, and aramid fibers [26,27]. They are used to reinforce the polymer matrix [28]. The fiber weave pattern can be classified into several categories of weaving methods, such as unidirectional and bidirectional methods, according to the shape and the distribution of the fabric. The bidirectional method exists in two orthogonal directions. Meanwhile, the unidirectional method has a single direction. Moreover, the bidirectional weaving method branches out into several shapes, including the satin weave, plain weave, and twill weave. The plain weave fibers achieve the most symmetrical texture distribution and the best mechanical characteristics when compared with the other weaving types [16].

Aromatic polyamides, polyester resins and epoxy resin are popular polymer matrix composite materials. Aromatic polyamides such as poly(ether sulfone), poly(ether ketone), and polyimide have a linear structure, interchain hydrogen bonds, and rigid aromatic rings in synthetic polymers. They are utilized in multiple applications because of their mechanically, chemically, and thermally stable characteristics. However, they cannot be processed by melting or dissolved in organic solvents. Polyester resins such as maleic anhydride are artificial types of resin fabricated by the interaction between polyhydric alcohols and dibasic organic acids. They are viscous and have pale-colored liquids. Although they are hydrophilic to glass fibers, they are toxic materials and are not suitable for molding many materials. Additionally, the final composite materials are weak against mechanical applications. Epoxy resin or polyepoxides are safe thermosetting polymer materials fabricated by the interaction between acidic hydroxy groups and epichlorohydrin. They are usually used as matrix materials for molding both carbon and glass fibers. Epoxy materials are well-adsorbed materials, with less shrinking and fewer non-volatile harmful gases. Therefore, they impregnate the porous fiber surfaces and increase the cohesion of the surface texture of the composite material. They also prevent severe chemical interactions and can improve the mechanical and the physical properties of the composite material, making it suitable for many different applications.

Carbon-fiber-reinforced polymers are well-known for their light weight, unique specific strength, good corrosion resistance, and good thermal stability [29]. Many industrial applications use carbon-fiber-reinforced polymers, such as piping systems [30,31], in gear assembly, and as conveys or aids [32]. On the other hand, glass-fiber-reinforced polymers are extensively available and inexpensive [16].

Additionally, they are widely used in several applications, such as: textiles, transportation, and sports equipment [33]. Nevertheless, there are some obstacles when using fiber-reinforced polymers. One of these limitations is their weak wear resistance [34,35]. Specifically, in some industrial applications, such as bushes, chutes lines, seals in pumps, vans, and the gears of mining equipment [36,37]. Therefore, it is necessary to enhance the wear resistance of the fiber-reinforced polymers in order to meet the requirements of industrial applications [38,39,40].

Researchers have investigated the behavior of the physical wear resistance of fiberglass reinforced with epoxy with and without nano-graphene from 0.5 to 1% wt as an additive filler precipitate. The study was applied using the pin-on-disk tribometer device to calculate the final coefficient of friction, added to the final wear resistance of the studied materials. The study concluded that the addition of the nano-graphene material led to an improved wear resistance of the examined materials and a lower wear rate for the surface of the examined materials, results which were supported by a surface structural characterization [41]. The fiber material orientation and epoxy as a matrix material are very critical parameters that affect material morphological, wear resistance, shear strength, ductility, and adhesive properties for special applications [42].

Previous studies investigated the wear characteristics of fiber-reinforced polymer through the Taguchi technique [15,43], which is concerned with electronic and electrical parts, such as the heavy voltage of electrical insulators, LEDs and brushes, as well as metal coatings, structural components, and components in the automotive industry [13,14]. The researchers used different sorts of fibers for reinforcement, such as glass, carbon, and Kevlar fibers; moreover, they selected epoxy HY 951 as a matrix. They applied the technique of a hand lay-up to fabricate substrates of PMCs. Furthermore, they performed a wear test using a pin-on-disk to determine the final wear resistance. Another study estimated the friction and wear resistance of fiber-reinforced polyimide composite samples to evaluate the wear characteristics of the examined material. They noted that the wear rate reduced when increasing the applied sliding speed, distance, and normal load. PMC laminate increases the FRPCs [44]. The researchers used various cutting fibers as reinforcement, with the surface of the PMCs acting as protective cover and decreasing the wear rate. Moreover, they increased the substrate thickness of the material, including glass, carbon, and aramid fibers. They selected a powder polyimide with a particle size of ˂75 mm and used a press-molding technique to produce a laminate of FRPCs. Finally, the study concluded that the wear resistance against dry sliding conditions was improved when merging polyimide into the cut fibers. Consequently, the coefficient of friction and the wear rate against three-body abrasion conditions were reduced when the polyimide was embedded into cut fibers. Meanwhile, the friction coefficient and wear rate of the glass fibers with polyimide against three-body abrasion wear conditions achieved the highest magnitude and a lower wear resistance. Another study investigated the influence of fiber type and fiber content on the wear characteristics of high-density polyethylene (HDPE) composites [45].

The researchers used different types of fibers, such as carbon, jute, basalt, and coconut fibers, as reinforcement materials. They also selected HDPE as a matrix material and carried out a melt blending technique via a twin screw in order to produce PMCs. They synthesized four compositions of PMCs with different weight fractions. They concluded that when the fiber concentration increases, the wear rate values are also increased. Additionally, the wear rate of the carbon-fiber-reinforced composites (HDPE/CF) exhibited higher values. Moreover, the increased sliding speed affected the final wear resistance of the investigated material.

The main objective of the current paper is to examine the structural and tribological properties of both the famous woven plain carbon and glass fibers through innovative geometrical alternatives against two stacking directions at horizontal and vertical orientation for bushing applications.

## 2. Materials and Methods

**Figure 1:**
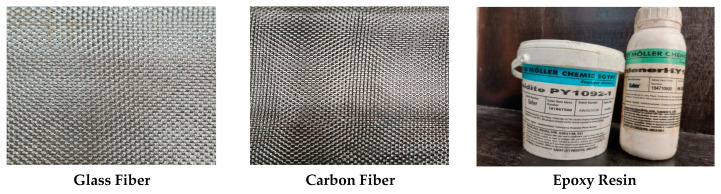
The selected glass fiber, carbon fiber, and epoxy resin materials.

Glass- and carbon-fiber reinforcement fabric materials are investigated in the current study. They were purchased at the Arab World for Financial Investments Company, Cairo, Egypt. Each fiber material comprises interlaced fiber yarns with two longitudinal and transverse directions. Detailed descriptions of the carbon and glass fiber fabric properties are described in Figure 1 and Table 1. The epoxy Araldite 1092 was used as the resin matrix material, as shown in Figure 1. It was provided by Alkoraem for the Chemical and Adhesion Materials Company, Cairo, Egypt.

Firstly, the layers (glass or carbon fibers) were set on an isolated flat and smooth surface where isolation was performed using plastic sheets as a release agent. After that, the matrix was prepared by adding 1:2 of the hardener material, grade HY1092, to the resin material, Araldite, grade PY1092. After mixing well, the resin solution was poured onto the surface of the first glass fabric or carbon fiber material until the surface of the fiber material was completely saturated by the resin. After that, the next glass or carbon fiber layer was added, respectively. The previous step was repeated for all the following layers until the required number of layers reached 40 layers. An insulator material was added to the surface of the last (glass or carbon fiber) material. Moreover, the total volume fraction of the carbon and glass fiber was 52% wt, while the total volume fraction of the epoxy resin was 48% wt of total matrix.

**Figure 2:**
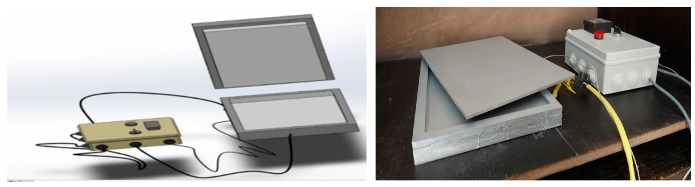
Solid works and design of compression molding.

**Figure 3:**
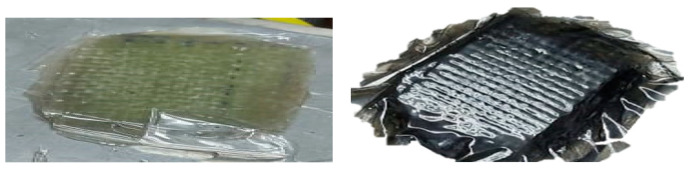
Glass and carbon composites after curing.

**Figure 4:**
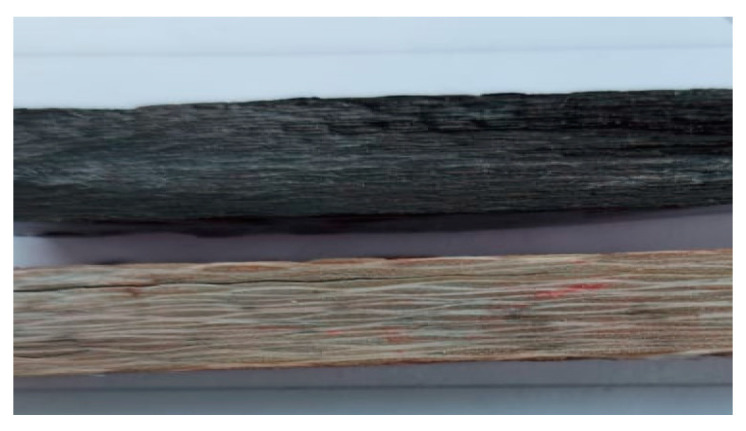
Rectangular glass and carbon composite samples.

The compression mold route was then initiated, and the prepared composite materials were adjusted for compression using the special electrical mold machine with dimensions of 300 × 200, as shown in Figure 2. The heat applied to the compressed composite materials was fixed at 55 °C for 30 min, and the applied compression load was fixed at 50 N, as shown in Figure 3. Finally, by using a low-speed saw, all samples were cut into pieces with 30 mm × 30 mm × 30 mm dimensions, as revealed in Figure 4, to be prepared for the following structural and tribological analyses [46,47,48,49].

Tribological analysis of the examined polymeric composite materials was carried out to investigate both the friction coefficient and the specific wear rate (SWR)at room temperature, using the pin-on-disk tribometer system. The wear system was made of a cylindrical-shape stainless steel alloy moving against several applied loads, ranging from 5, 10, 15, and 20 Newtons. The speed of motion was fixed at a steady speed of 0.520 m/s to achieve a final sliding distance of 1000 m against the applied loads. The test was applied without any lubricant, and the friction coefficient was recorded at the same time as the application of the tribological analysis [38]. The weight of the investigated samples was measured, using a four-digit balance with an accuracy of up to 0.0001 g, before and after the application of the tribological analysis to calculate the final specific wear rate (SWR) value, according to the following equation:

(1) $$\left(\mathrm{SWR})=\lceil \frac{\Delta w}{\mathrm{Ff}.\rho .V.t}\rceil ({\mathrm{cm}}^{3}/N.m\right)$$

where, ∆w represents the weight loss of sample after the test, per gram; Ff represents the friction force of machine, per N; ρ represents the density of sample, per cm^3^/g; V is the speed velocity, per (m/s); and t is the duration time of test, per second.

To achieve the best accuracy for each experiment, the oscillating bark disk must be polished and cleaned by coarse sanding paper (p320), followed by sandpaper (p1000), before every single test with high, pure ethanol alcohol. It must then be dried to eliminate any residual radioactive precipitates. The morphological characterization of the investigated material [50,51,52] and the final wear resistance of the composite polymeric materials of the investigated samples were conducted using the SEM instrument before and after the application of the tribological wear test.

## 3. Results

### 3.1. Structural and Tribological Characterization for Glass Fiber Composite Material

**Figure 5:**
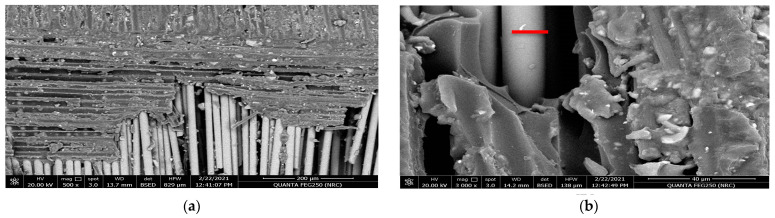
SEM for the resin molded horizontally in the stacking direction of the glass fibers before the tribological analysis: (**a**) magnification 500× and (**b**) magnification 3000×.

**Figure 6:**
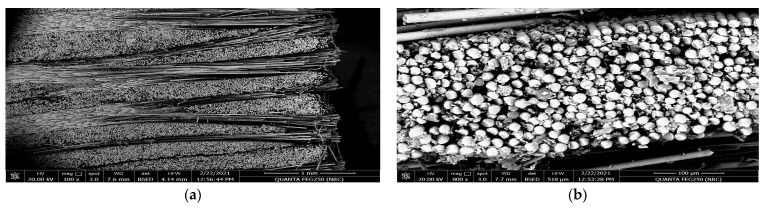
SEM for the resin molded vertically by the stacking direction of the glass fibers before the tribological analysis: (**a**) magnification 100× and (**b**) magnification 800×.

Figure 5a,b and Figure 6a,b reveal the impact of the geometrical alternatives for fiber directions on the surface morphology of the glass fiber composite material before applying the tribological analysis. As a hardener material, the resin epoxy was molded horizontally and vertically to the stacking direction of the plain weave glass fiber [42]. Figure 5a represents the SEM for the surface of the glass fiber in the horizontal direction at magnification 500×. It is obvious that the resin was not absorbed by or merged well with the fiber glass; this is demonstrated well at a higher magnification 3000× in Figure 5b. Additionally, from Figure 5b, the diameter of a glass fiber unit can be estimated to be about 16.14 µm. Figure 6a,b represent the SEM at magnifications 100× and 800× for the surface of the glass fiber when the resin was molded in a direction vertical to the glass fiber. It can be noted that the fiber glass repelled the molded epoxy resin, as the fiber glass appears more like a hydrophobic material. It is clear that at a the higher magnification, 800×, the poured resin material takes a droplet shape and appears more ballistic because the surface tension between the resin and the fiber glass is very weak, according to the increased adhesion between the resin molecules and the weak surface tension of the surface of the fiber glass material.

#### Tribological Analysis of the Glass Fiber Composite Material

**Figure 7:**
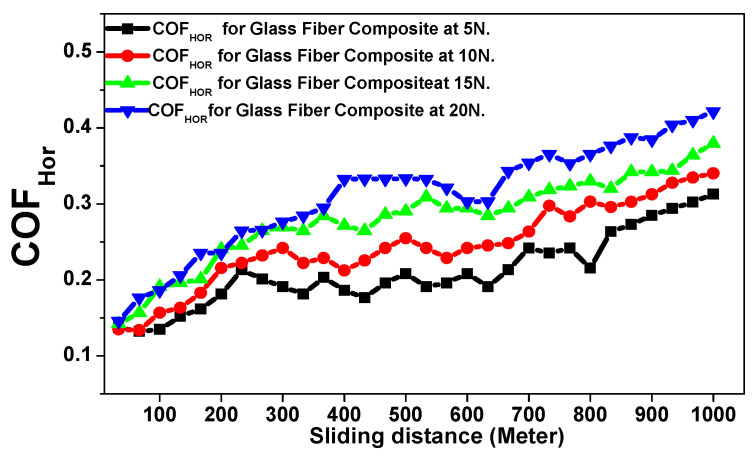
Friction coefficient versus sliding distance (meters) of the glass fiber composite material against the horizontal direction as a function of the applied loads.

**Figure 8:**
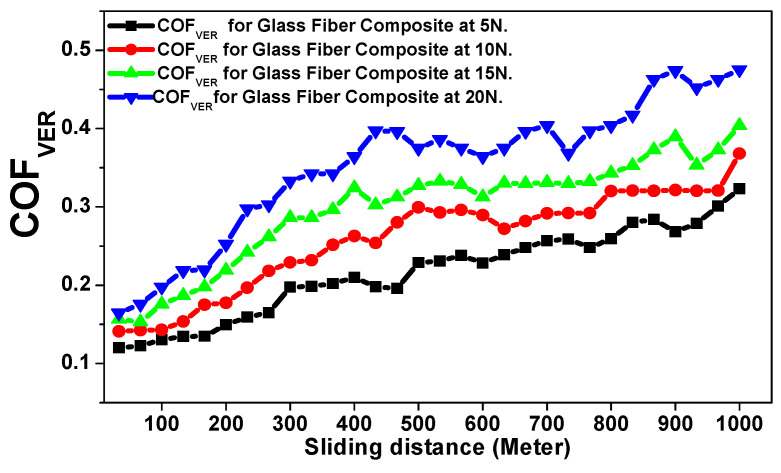
Friction coefficient versus sliding distance (meters) of the glass fiber composite material against the vertical direction as a function of the applied loads.

**Figure 9:**
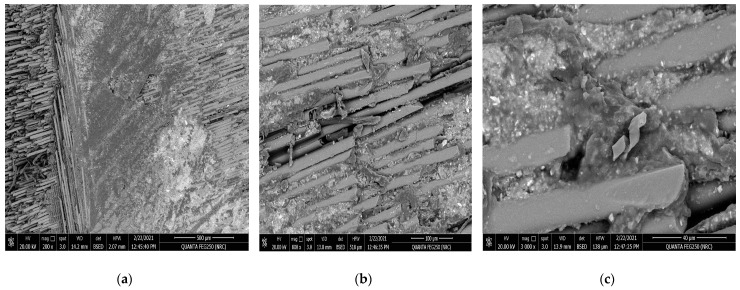
SEM for the resin molded horizontally in the stacking direction of the glass fibers after tribological analysis: (**a**) magnification 200×, (**b**) magnification 800×, and (**c**) magnification 3000×.

**Figure 10:**
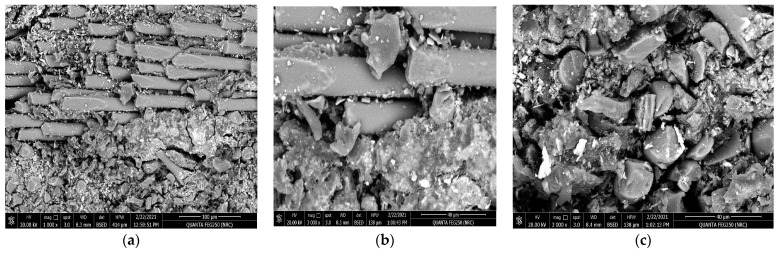
SEM for the resin molded vertically in the stacking direction of the glass fibers after tribological analysis, (**a**) magnification 1000×, (**b**) magnification 3000×, and (**c**) magnification 3000×.

The abrasive tribological properties of the glass fiber composite material were investigated in two directions (horizontal and vertical)relative to the glass fiber stacking direction against applied loads of 5, 10, 15, and 20 N to estimate the final wear resistance of the investigated polymer composite material [17,18,19,20,21,22]. Figure 7 and Figure 8 reveal the behavior of the friction coefficient of the glass fiber material in the horizontal and the vertical directions as a function of the total sliding distance against several applied loads. The rotating steel bark disk speed was fixed at 0.520 m/sec. Table 2 represents the tribological data analysis of the investigated composite material [17,18,19,20,21,22]. Furthermore, Figure 9a–c and Figure 10a–c represent the SEM of the final surface morphology of the worn surfaces at the end of the tribological tests at the highest applied load 20 N against the two orientations.

### 3.2. Structural and Tribological Charactrizationof CarbonFiber Composite Materials

**Figure 11:**
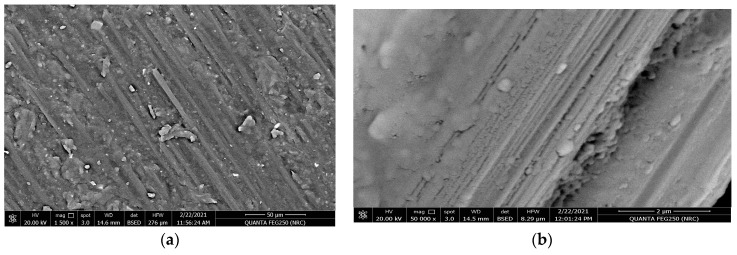
SEM for the resin molded horizontally in the stacking direction of the carbon fibers before the tribological analysis: (**a**)magnification 1500× and (**b**) magnification 50,000×.

**Figure 12:**
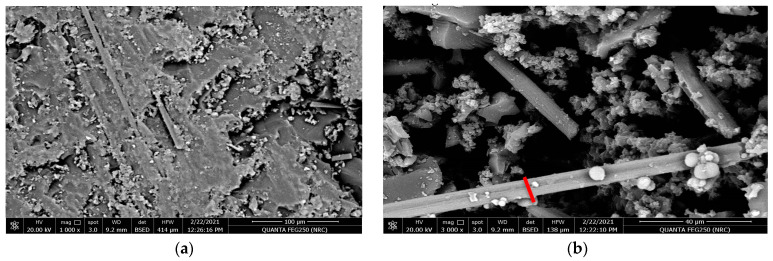
SEM for the resin molded vertically in the stacking direction of the carbon before the tribological analysis: (**a**)magnification 1000× and (**b**) magnification 3000×.

Figure 11a,b and Figure 12a,b represent the surface morphology of the carbon fiber composite material when the resin was molded horizontally and vertically in the carbon fiber stacking direction before applying the tribological analysis. Figure 11a,b represent the surface texture of the carbon fiber in the horizontal direction at magnifications of 1500× and 50,000×. It is obvious that the resin was highly absorbed and is very integrated, highly homogeneous, and more streamlined with the carbon fibers, which is explained well at the higher magnification of 500,000× by Figure 11b. Figure 12a,b represent the SEM of the surface of the carbon fiber composite material in the vertical direction at magnifications of 1000× and 3000× before the tribological analysis. It is clear from this texture that the resin merged and gathered in the form of blocks around the carbon fiber. Additionally, the diameter of the carbon fiber is up to 10.9 µm, which is approximately two-thirds of the diameter of the glass fiber material.

#### Tribological Analysis of the Carbon Fiber Composite Material

**Figure 13:**
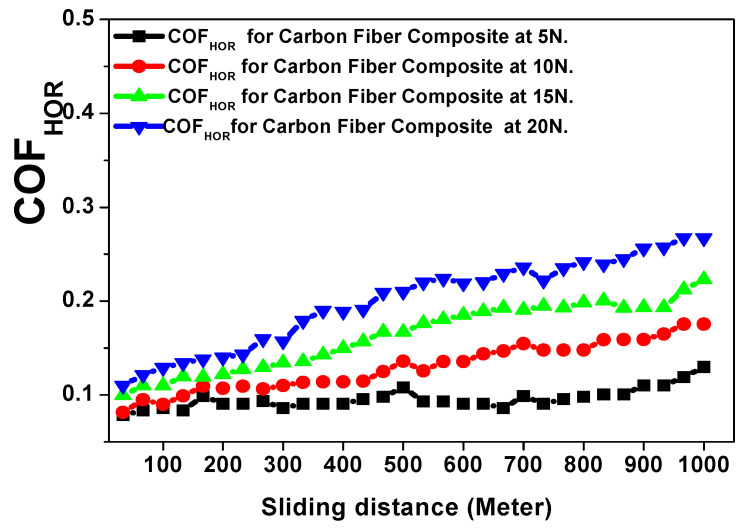
Friction coefficient versus sliding distance (meters) of the carbon fiber composite material against the horizontal direction as a function of the applied loads.

**Figure 14:**
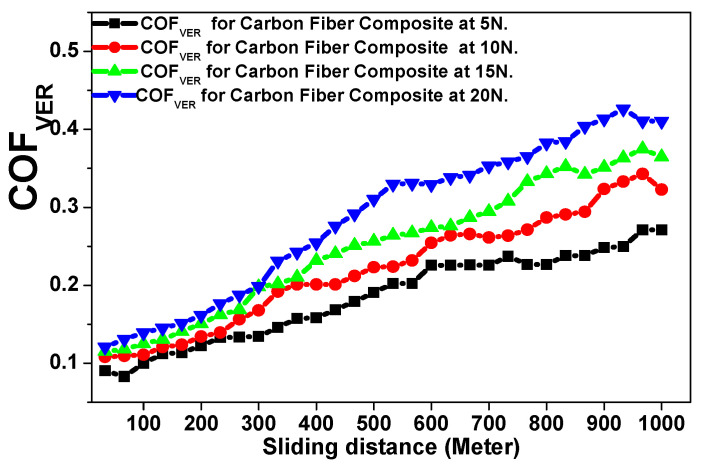
Friction coefficient versus sliding distance (meters) of the carbon fiber composite material against the vertical direction as a function of the applied loads.

**Figure 15:**
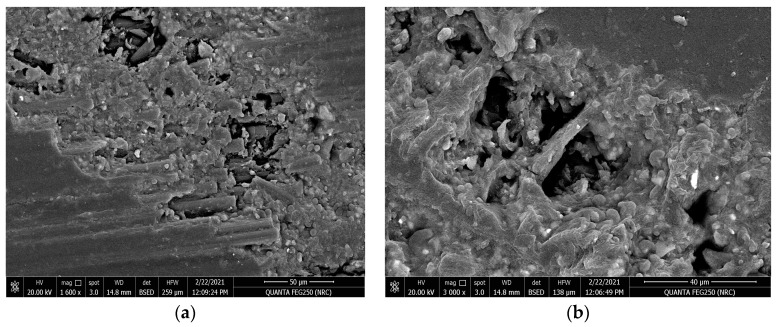
SEM for the resin molded horizontally inthe stacking direction of the carbon fibers direction after tribological analysis: (**a**) magnification 1600× and (**b**) magnification 3000×.

**Figure 16:**
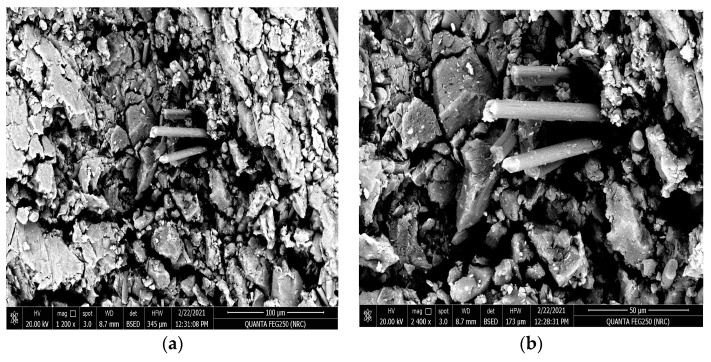
SEM for the resin molded vertically in the stacking direction of the carbon fibers after tribological analysis, (**a**) magnification 1200× and (**b**) magnification 2400×.

The abrasive tribological analysis of the Carbon Fiber composite material was performed using the same applied tribological conditions for the glass fiber composite material. Figure 13 and Figure 14 represent the behavior of the friction coefficient of the carbon fiber composite material in both the horizontal and vertical directions as function of the total sliding distance against the 5, 10, 15, and 20 N applied loads [17,18,19,20,21,22]. Table 3 represents the tribological data analysis of the investigated carbon fiber composite as a function of the applied loads. Finally, Figure 15 and Figure 16 represent the SEM of the final morphology of the worn surface at the end of the tribological tests at the highest applied load, 20 N in the horizontal and vertical directions.

## 4. Discussion

The tribological analyses for the investigated materials will be analyzed by explaining the behavior of the friction coefficient and the final specific wear rate (SWR) per (cm^3^/N.m) for the investigated materials in both the horizontal and vertical orientations. Figure 7 indicates the behavior of the friction coefficient against applied loads of 5, 10, 15, and 20 N as a function of the sliding distance for the glass fiber composite material against the horizontal stacking direction. It is clear that the friction coefficient increases directly with the increased sliding distance and the increased applied loads [17,18,19,20,21,22]. However, this relationship is not continually a regular, smoothly shaped linear relationship. As is shown, there are many random fluctuations occurring on the coefficient of friction curve due to the lack of merging with the resin by the stacking glass fiber, as shown in Figure 5a,b.The behavior of the COF was ensured to fit well with the obtained results, as represented in Table 1, where the lowest mean value of the friction coefficient and the SWR (0.2022 ± 0.0048 and (3.793 ± 0.0091) × 10^−10^ (cm^3^/N.m))were achieved at an applied load of 5 N. Meanwhile, the highest mean value of the friction coefficient and (0.3325 ± 0.0071 and (8.3804 ± 0.0180) × 10^−10^ (cm^3^/N.m))were achieved at an applied load of 20 N.

The impact of the tribological test on the horizontal resin to the fiber glass stacking direction is obvious in Figure 9a–c. The SEM measurements were applied at several magnifications (200×, 800×, and 3000×) for the surface morphology of the resin molded horizontally in the stacking direction of the glass fibers after tribological analysis at an applied load of 20 N. The direction of tribological friction is clear in Figure 9a. Moreover, Figure 9b,c show the existence of very fine and brittle residual precipitates and fragments remaining at the surface of the fiber glass composite sample at the end of tribological abrasive test at higher SEM magnifications of 800× and 3000×.

Figure 8 reveals the behavior of the friction coefficient versus sliding distance (meters) of the glass fiber composite material in the vertical direction as a function of the applied loads. It is clear that the material wear resistance is weaker at a vertical orientation compared with the horizontal direction, where both the obtained mean value for the friction coefficient and the SWR at 5 N (0.2120 ± 0.0056 and (5.9782 ± 0.0148) × 10^−10^ (cm^3^/N.m)) and at 20 N (0.3751 ± 0.0088 and (9.6739 ± 0.0228) × 10^−10^ (cm^3^/N.m)) are higher than the values obtained for a horizontal direction of the resin to the stacking direction of the glass fiber. The impact of the tribological wear at an applied load of 20 N on the investigated glass fiber sample with the resin vertically oriented to the stacking direction of the glass fibers is represented by Figure 10a–c, where, the SEM shows the surface morphology after the wear test at multiple magnifications of 1000× and 3000×. The obtained surface morphology agrees well with the previously mentioned fact that the resin is not completely merged with fiber glass, as shown in Figure 6a,b. Moreover, Figure 10b,c indicate and confirm that the friction has been increased according to the increased percentage of fragments and debris surrounding the fiber glass. These increased precipitates act as an additional parameter, accelerating the tribological process and increasing the final specific wear rate of the corroded material when compared with the resin applied in a parallel direction to the stacking direction of the glass fiber [17,18,19,20,21,22].

The effect of the tribological wear measurements on the carbon fiber composite material against both the horizontal and vertical orientations of the resin to the carbon fiber direction are explained by discussing the behavior of the friction coefficient and wear volume loss represented in Figure 13, Figure 14, Figure 15 and Figure 16 and Table 3. The behavior of the friction coefficient versus the sliding distance (meters) of the carbon fiber composite material in the horizontal direction as function of the applied load is represented in Figure 13. The behavior is smoother and quieter compared with the previously mentioned behavior of the glass fiber composite material at the same orientation, with a lower mean friction coefficient and SWR achieved at 5 N (0.0931 ± 0.0010 and (1.6304 ± 0.0019) × 10^−10^ (cm^3^/N.m)) and at 20 N (0.2145 ± 0.004 and (4.3369 ± 0.0095) × 10^−10^ (cm^3^/N.m)). The SEM in Figure 15a,b represent two magnifications, 1600× and 3000×, for the surface morphology of the composite materials when the resin is molded horizontally to the stacking direction of the carbon fibers after a tribological analysis against an applied load of 20 N.The influence of the wear test in the direction of friction and the texture of the resulting pitting on the corroded surface are shown in Figure 15a at a magnification 1600×. Moreover, at a higher magnification of 3000×, it is represented in Figure 15b; it is clear that the high saturation and merging between the resin and carbon fiber caused an agglomeration of debris around the carbon fiber, leading to a lower specific wear rate of the corroded surface at the end of the wear test compared with the glass fiber composite material.

The behavior of the friction coefficient versus the sliding distance (meters) of the carbon fiber composite material as a function of the applied loads against the vertical direction is represented in Figure 14. The friction coefficient is increased by the vertical direction compared with the horizontal direction of the same composite material, with an obtained mean friction coefficient and SWR specific wear rate at 5 N (0.2061 ± 0.0056 and (3.6956 ± 0.0107) × 10^−10^ (cm^3^/N.m)) and at 20 N (0.3751 ± 0.0099 and (6.5217 ± 0.0203) × 10^−10^ (cm^3^/N.m)). Moreover, the final mean friction coefficient and specific wear rate for the carbon fiber composite material are still lower than those obtained for the glass fiber composite material.

The following Figure 16a,b show the SEM at the magnifications 1200× and 2400× for the surface texture of the carbon fiber composite material when the resin is molded vertically to stacking direction of the carbon fibers after the tribological analysis at an applied load of 20 N. It is clear that the worn surface of the investigated material shows the existence of larger pitting and more physical corrosion compared with the previous horizontal orientation after the applied wear test. However, the amount of the worn material under the same applied tribological condition is still lower than that obtained for the glass fiber composite material, which is compatible with the obtained results [17,18,19,20,21,22].

## 5. Conclusions

The article introduced the structural and tribological properties of carbon and glass fiber composite materials at two different geometrical alternatives through two stacking directions to investigate their capability for bushing applications. The detailed observations were checked and analyzed before and after a wear test to clarify the change in structural characteristics against the applied wear conditions. The study deduced that the behavior of the friction coefficient and the specific wear rate of the corroded material are due to the final wear resistance of the tested material, the surface texture of the investigated the material, nature of the physical and chemical interactions between the composite additives, or the fiber material which makes the final polymer. Moreover, the positioning of the samples, including the orientation of the resin to the fiber material against the applied tribological wear conditions, control the final wear resistance of the investigated composite material, which is critical for the safety of bushing applications.

## Figures and Tables

**Table 1:** Detailed description of carbon-and glass-fiber fabrics.

Product	Glass Fiber	Carbon Fiber
Warp Raw	2 k, multifilament continuous	3 k, multifilament continuous
Filling Raw	2 k, multifilament continuous	3 k, multifilament continuous
Weave Pattern	Plain	Plain
Surface Density	320 g/m^2^	200 g/m^2^
Dressing Agents	Saline-coated/Volans-treated after heat cleaning	None

**Table 2:** Tribological analysis of the glass fiber composite material.

Material	Applied Load (N)	Friction Coefficient Mean	Specific Wear Rate (cm^3^/N.m)
Horizontal Glass Fiber Composite	5	0.2022 ± 0.0048	(3.7934 ± 0.0091) × 10^−10^
	10	0.2419 ± 0.0055	(5.5217 ± 0.0126) × 10^−10^
	15	0.2882 ± 0.0058	(6.5326 ± 0.0131) × 10^−10^
	20	0.3325 ± 0.0071	(8.3804 ± 0.0180) × 10^−10^
Vertical Glass Fiber Composite	5	0.2120 ± 0.0056	(5.9782 ± 0.0148) × 10^−10^
	10	0.2810 ± 0.0063	(7.500 ± 0.0169) × 10^−10^
	15	0.3258 ± 0.0068	(8.1521 ± 0.0172) × 10^−10^
	20	0.3751 ± 0.0088	(9.6739 ± 0.0228) × 10^−10^

**Table 3:** Tribological analysis of the carbon fiber composite.

Material	Applied Load (N)	Friction Coefficient Mean	Specific Wear Rate (cm^3^/N.m)
Horizontal Carbon Fiber composite	5	0.0931 ± 0.0010	(1.6304 ± 0.0019) × 10^−10^
	10	0.1306 ± 0.0025	(2.9021 ± 0.0057) × 10^−10^
	15	0.1718 ± 0.0035	(3.2717 ± 0.0067) × 10^−10^
	20	0.2145 ± 0.004	(4.3369 ± 0.0095) × 10^−10^
Vertical Carbon Fiber Composite	5	0.2061 ± 0.0056	(3.6956 ± 0.0107) × 10^−10^
	10	0.2810 ± 0.0072	(4.7826 ± 0.0155) × 10^−10^
	15	0.3258 ± 0.0083	(5.6521 ± 0.0181) × 10^−10^
	20	0.3751 ± 0.0099	(6.5217 ± 0.0203) × 10^−10^

## Data Availability

Data are available upon request.
